# Clinical course of pharyngotonsillitis with group A streptococcus treated with different penicillin V strategies, divided in groups of Centor Score 3 and 4: a prospective study in primary care

**DOI:** 10.1186/s12879-022-07830-4

**Published:** 2022-11-11

**Authors:** David Tell, Mia Tyrstrup, Charlotta Edlund, Karin Rystedt, Gunilla Skoog Ståhlgren, Pär-Daniel Sundvall, Katarina Hedin

**Affiliations:** 1 Futurum, Region Jönköping County, and Department of Health, Medicine and Caring Sciences, Linköping University, Linköping, Sweden; 2 Department of Clinical Sciences in Malmö, Family Medicine, Lund University, Lund, Sweden; 3 Unit for Antibiotics and Infection Control, The Public Health Agency of Sweden, Solna, Sweden; 4 General Practice/Family Medicine, School of Public Health and Community Medicine, Institute of Medicine, Sahlgrenska Academy, University of Gothenburg, Gothenburg, Sweden; 5 Research, Education, Development & Innovation, Primary Health Care, Region Västra Götaland, Gothenburg, Sweden; 6 Centre for Antibiotic Resistance Research (CARe) at University of Gothenburg, Gothenburg, Sweden

**Keywords:** Pharyngotonsillitis, Centor Score 3 and 4, Phenoxymethylpenicillin, Primary care

## Abstract

**Background:**

Sore throat is a common reason for prescribing antibiotics in primary care, and 10 days of treatment is recommended for patients with pharyngotonsillitis with group A streptococcus (GAS). Our group recently showed that penicillin V (PcV) four times daily for 5 days was non-inferior in clinical outcome to PcV three times daily for 10 days. This study compares duration, intensity of symptoms, and side effects in patients with a Centor Score (CS) of 3 or 4 respectively, after treatment with PcV for 5 or 10 days and evaluates whether all patients with pharyngotonsillitis with a CS of 3 or 4 should be treated for 5 days or if severity of symptoms or CS suggest a longer treatment period.

**Method:**

Data on symptoms and recovery from patient diaries from 433 patients included in a RCT comparing PcV 800 mg × 4 for 5 days or PcV 1 g × 3 for 10 days was used. Patients six years and older with CS-3 or CS-4 and positive rapid antigen detection test for GAS-infection were grouped based on CS and randomized treatment. Comparisons for categorical variables were made with Pearson’s chi-squared test or Fisher’s exact test. Continuous variables were compared with the Mann–Whitney U test.

**Results:**

Patients with CS-3 as well as patients with CS-4 who received PcV 800 mg × 4 for 5 days self-reported that they recovered earlier compared to patients with CS-3 or CS-4 who received treatment with PcV 1 g × 3 for 10 days. In addition, the throat pain as single symptom was relieved 1 day earlier in patients with CS-4 and 5 days of treatment compared to patients with CS-4 and 10 days of treatment. No differences in side effects between the groups were found.

**Conclusion:**

Intense treatment with PcV four times a day for 5 days seems clinically beneficial and strengthens the suggestion that the 4-dose regimen with 800 mg PcV for 5 days may be the future treatment strategy for GAS positive pharyngotonsillitis irrespectively of CS-3 or CS-4.

*Trail registration* ClinicalTrials.gov ID: NCT02712307 (3 April 2016).

## Background

Antibiotic resistance is of great concern and the use of antibiotics is the major force driving resistance [1]. The number of consultations and the prescribing rate of antibiotics for respiratory tract infections differs between countries [2]. A common reason for prescribing antibiotics is sore throat. In 2013, approximately 18 prescriptions per 1000 patients were due to sore throat, accounting for about 11% of all antibiotic prescriptions in primary health care in Sweden [3].

The European Society of Clinical Microbiology and Infectious Diseases (ESCMID) Sore Throat Guideline Group [4] and the Swedish national guidelines [5] recommend using Centor criteria (fever, tender cervical lymph nodes, coatings of the tonsils, and lack of cough) [6] to identify patients who have a higher likelihood of group A streptococcus (GAS) infection. This group (i.e., CS-3 and CS-4) is more likely to benefit from antimicrobial treatment [4, 5]. Patients with CS-3 or CS-4 should be offered antibiotic treatment if they have a positive rapid antigen detection test (RADT) for GAS [4–6].

A Cochrane report from 2012 recommends 10 days treatment with penicillin [7], and a review from 2020 concluded that long-course phenoxymethylpenicillin (PcV) should remain as the first line antibiotic for the treatment of patients with streptococcal pharyngitis. In this review, most studies compared long-term antibiotic therapy (10 days with PcV) with short-term broad spectrum antibiotics; the authors of the review called for trials that assess the effectiveness of different doses and lengths of PcV treatment [8]. There has also been a request for studies measuring the impact of antibiotics on severity of symptoms in pharyngotonsillitis [9]. Our group recently showed that PcV 800 mg four times daily for 5 days was non-inferior in clinical outcome to PcV 1 g three times daily for 10 days in patients with pharyngotonsillitis, with a CS of 3 or 4 with confirmed presence of GAS. The clinical cure rate appeared to be lower in patients with CS 4 receiving the shorter treatment [10]. However, it is currently unknown whether the patient judgement of severity of symptoms in combination with level of CS suggest that a longer treatment period is needed.

This study compares duration and intensity of symptoms based on patient’s diaries before and after initiation of treatment with PcV as well as the occurrence of side effects after treatment with PcV for 5 or 10 days in patients six years and older with acute pharyngotonsillitis with a CS of 3 or 4 and positive for GAS.

## Method

This study is based on data from a study described in a previously published paper [10]. In summary, 433 patients six years and older with acute pharyngotonsillitis, a CS of 3 or 4 and positive RADT for GAS were randomized to either PcV 800 mg × 4 for 5 days or PcV 1 g × 3 for 10 days. Patients were excluded if they had signs of serious illness, had hypersensitivity to penicillin, were receiving immunomodulation treatment corresponding to at least 15 mg of prednisolone, had received antibiotics for pharyngotonsillitis in the past month, or had received any antibiotic treatment within 72 h before inclusion [10]. The patients (or guardian) registered symptoms, intensity of symptoms, and side effects in a diary until a follow-up visit 5 to 7 days after end of treatment. During a follow-up telephone call one month after completion of antibiotic treatment, regional study nurses asked the participants if they were experiencing throat symptoms, relapses, or new tonsillitis, complications, and adverse events. Throat swabs for RADT and culture for GAS identification were performed at the inclusion visit and at the follow-up visit. The same procedures were performed in the groups. Patients were recruited from 17 primary healthcare centres in urban and rural regions of Sweden.

GAS isolates were sent to the local microbiological laboratory for culturing and then to the Public Health Agency of Sweden. At the Public Health Agency of Sweden, GAS isolates from patients without bacteriological eradication at the follow-up visit and with an available isolate from the inclusion visit were *emm* typed [11] so pairwise relatedness within patients could be determined.

In the present study, the 422 patients from the modified intention to treat population were included [10], except two patients who did not have a CS of 3 or 4, so the final sample was 420 patients. These patients were grouped based on CS (CS-3 or CS-4) and randomized treatment (PcV 800 mg × 4 for 5 days or PcV 1 g × 3 for 10 days). We used data from the patient diaries to assess time to self-reported recovery from infection, return to work/school or equivalent, relief of fever, number of days using painkillers, and throat symptoms (no symptoms, mild, moderate, or severe symptoms). In addition, we analysed occurrence of side effects and number of days with reported side effects such as diarrhoea, nausea/vomiting, vaginal itching or discharge, and rash. We compared the number of days of the above variables between the four groups.

Two of the authors (DT, KH) discussed and classified the patients with incomplete diaries, missing data, and deviations in answers. Patients with incomplete registrations on the question ‘Do you consider yourself or your child recovered from the current infection’ (82/420) were considered recovered when they had absence of fever (< 37.6 °C) and no sore throat reported in the diary. We defined no sore throat symptoms as no or mild reported sore throat symptoms [12, 13]. According to this definition, another 27 patients were eligible for analysis, resulting in 365 patients available for analysis.

When analysing specific side effects, patients were excluded if no data were registered in the diary or if the patient reported side effects on the day of inclusion. Of the 420 patients, 34 did not answer the question about adverse events (diarrhoea, nausea or vomiting, and rash) at all. Of the 269 women, 37 did not answer the question regarding vaginal itching or discharges.

### Statistical methods

Categorical variables were presented as numbers and percentages, and comparisons between groups were made with Pearson’s chi-squared test or Fisher’s exact test. Continuous variables were presented, unless stated otherwise, as median, minimum, and maximum and were compared with the Mann–Whitney U test.

We analysed time to self-reported recovery, time to relief of the single symptom sore throat and the symptom fever, days using painkillers, absence from work or school between the four groups using the log rank test. The two groups with CS-3 were compared and the same comparisons were made between the groups with CS-4. Data were censored on the first day of symptom free recording for the variables self-reported recovery, sore throat, fever and pain, if the symptom relief persisted at least two days. Safety was presented using descriptive statistics. We set the level of significance to 5%, two sided. We performed all analyses using SPSS statistics version 27.0.1.0.

## Result

The 420 patients were divided into four groups: CS-3 1 g × 3 10 days, CS-3 800 mg × 4 5 days, CS-4 1 g × 3 10 days, and CS-4 800 mg × 4 5 days. Baseline data were comparable between the groups except for severity of throat pain, which showed a statistically significant difference between the four groups (Table 1). Compared with patients with CS-3, more patients with CS-4 rated their throat pain as severe (p = 0.0017).

**Table 1:** Baseline characteristics for the population (n = 420) divided into groups based on Centor Score (3 or 4) and given treatment (Penicillin V 800 mg × 4 for 5 days or Penicillin V 1 g × 3 for 10 days) according to the physicians report

	CS-3 1 g × 3 10 days n = 104	CS-3 800 mg × 4 5 days n = 104	CS-4 1 g × 3 10 days n = 105	CS-4 800 mg × 4 5 days n = 107	p-value*
Women	71 (68)	65 (62)	61 (59)	72 (67)	0.39
Age in years: median (range)	30 (3–67)	30 (6–73)	31 (7–63)	30 (7–57)	0.29
Age group					
≤ 11	13 (13)	19 (18)	14 (13)	19 (18)	
12–17	10 (10)	10 (10)	13 (12)	13 (12)	
≥ 18	81 (78)	75 (72)	78 (74)	81 (76)	
Weight (kg): median (range)	68 (12.5–130)	65(18–114)	70 (21–126)	68 (27–116)	0.19
Smoker	9 (9)	8 (8)	5 (5)	11 (10)	0.63
Fever ≥ 38.5 °C	56 (54)	50 (48)	105 (100)	107 (100)	
Tender lymph nodes	83(80)	92 (89)	104 (100)	107 (100)	
Coating of the tonsils	80 (77)	74 (71)	105 (100)	107 (100)	
No cough	80 (89)	74 (92)	105 (100)	107 (100)	
Positive culture for GAS at inclusion visit	84 (81)	92 (89)	91 (87)	99 (92)	0.13
Days with throat pain before inclusion visit, median (range)	3 (1–15)	3 (1–14)	3 (1–30)	3 (1–13)	0.44
Throat pain according to the patient					
Mild	2 (2)	5 (5)	5 (5)	3 (3)	
Moderate	49 (47)	46 (44)	31 (30)	33 (31)	0.048
Severe	53 (51)	53 (51)	69 (65)	71 (66)	
General condition according to the physician					
Mildly affected	41 (39)	35 (34)	28 (27)	30 (28)	
Moderately affected	63 (61)	69 (66)	77 (73)	77 (72)	0.18
Impact of the infection					
Ability to eat and drink	89 (86)	86 (83)	96 (91)	96 (90)	0.22
Sleep	78 (75)	83 (81)	85 (80)	85 (80)	0.75
General condition	86 (83)	92 (89)	91 (87)	99 (92)	0.30
Daily activity	88 (85)	86 (83)	94 (80)	96 (90)	0.65
Days (median)	2	2	2	2	0.23
Tonsillectomized	4 (4)	3 (3)	3 (3)	2 (2)	0.86
> 3 antibiotic-treated tonsillitis last year	3 (3)	1 (1)	1 (1)	3 (3)	0.52
Children < 18 years in the household	74 (71)	79 (76)	77 (73)	81 (76)	0.62
Ongoing throat infection in family or related	30 (29)	24 (23)	32 (31)	35 (33)	0.60

Values are numbers (percentages) unless stated otherwise

*Comparison between the four groups

**Fig. 1:**
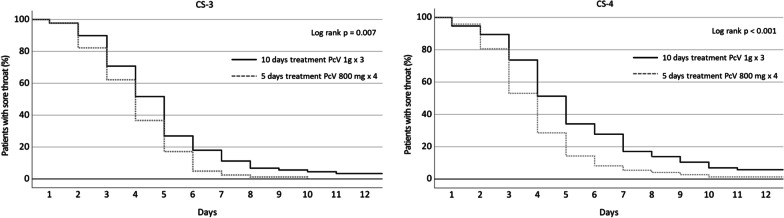
Time to first day of self-reported recovery according to patients’ diaries for patients with Centor Score of 3 or 4 and 5 or 10 days with PcV treatment, respectively

There was no significant difference in missing data regarding self-reported recovery or sore throat as a single symptom in the diaries between the four groups (p = 0.70 and p = 0.50). According to the patients’ diaries, time to first day of self-reported recovery was significantly shorter in the 5-day treatment group compared with the 10-day treatment group, irrespective if the patient had CS-3 (p = 0.007) or CS-4 (p < 0.001) (Fig. 1). The median number of days to recovery was 4 (1–18) days in the CS-3 10-day group, 3 days (1–10) in the CS-3 5-day group, 4 days (1–18) in the CS-4 10-day group, and 3 days (1–13) in the CS-4 5-day group.

**Fig. 2:**
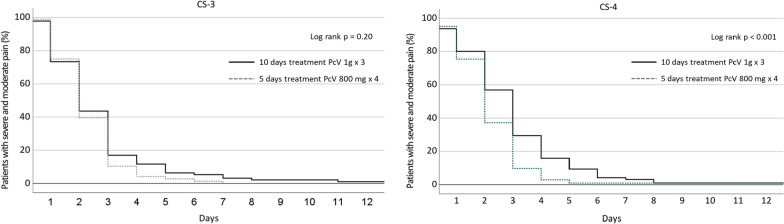
Time to first day of reporting mild or no pain in the throat according to patients’ diaries for patients with Centor Score 3 or 4 and 5 or 10 days with PcV treatment

The throat pain as single symptom relieved earlier among patients in the CS-4 5-day treatment group compared with the CS-4 10-day treatment group (p < 0.001); however, for those with CS-3, there were no significant differences in number of days to relief of throat pain between the treatment groups (p = 0.20) (Fig. 2).

On the day of inclusion (day 0), 73% of the patients in the CS-4 5-day group, 67% in the CS-4 10-day group, 59% in the CS-3 5-day group, and 60% in the CS-3 10-day group rated their throat pain as severe (Table 2). The median time with reported severe and moderate pain was two days in all groups except in CS-4 10-day group, where the median time with severe or moderate pain was 3 days.

**Table 2:** Proportion (%) of patients with severe, moderate, mild or no throat pain according to patients’ diaries for the 420 patients divided into groups based on Centor Score (3 or 4) and given treatment (Penicillin V 800 mg × 4 for 5 days or Penicillin V 1 g × 3 for 10 days)

Day	CS-3 1 g × 3, 10 days n = 104				CS-3 800 mg × 4, 5 days n = 104				CS-4 1 g × 3, 10 days n = 105				CS-4 800 mg × 4, 5 days n = 107			
	Severe	Moderate	Mild	No	Severe	Moderate	Mild	No	Severe	Moderate	Mild	No	Severe	Moderate	Mild	No
0	60	36	3		59	33	2		67	18	6		73	21	6	
1	29	45	23	3	31	41	24	4	48	32	18	2	26	50	24	1
2	9	36	38	17	7	30	43	19	18	39	32	12	4	34	48	14
3	5	14	48	33	3	8	50	39	9	21	39	31	1	10	44	45
4	2	10	35	53	2	1	32	65	2	14	36	48	0	3	28	69
5	4	3	25	68	1	1	14	84	0	10	28	63	0	1	20	79
6	2	4	18	76	0	3	8	89	1	3	23	72	0	3	12	86
7	1	3	12	84	0	1	13	86	0	3	11	87	1	1	16	82
8	1	1	9	90	1	3	10	86	0	1	11	88	1	5	13	80
9	1	1	8	91	0	4	10	86	0	2	4	94	0	10	14	77
10	1	1	7	91	0	5	8	88	0	2	4	94	2	4	13	81

At the inclusion visit, 84% in CS-4 5-day group and 86% in CS-4 10-day group used pain relievers. The proportion of patients with CS-4 taking pain relievers decreased faster in the group taking PcV four times a day compared with patients taking PcV three times a day (p < 0.001), and 76% in CS-3 5-days group and 72% in the CS-3 10-day group used pain relievers at inclusion (p = 0.14).

There was no difference between the groups in number of days to relief of fever recorded in patients’ diaries (CS-3 5-days vs. 10-days, p = 0.62; CS-4 5-days vs. 10-days, p = 0.67) and no differences between the groups regarding the return to work or school (CS-3, p = 0.90, CS-4, p = 0.70). There were no differences in days of self-reported recovery and in severity of sore throat in children (6– ≤ 11 years) regardless of CS and treatment regimen.

After self-reported recovery from infection, 5% reported recurrence of sore throat-related illness. Table 3 presents the number of patients with new symptoms, days to recurrence of sore throat-related symptoms, duration of sore throat-related symptoms, bacterial eradication, and new acute pharyngotonsillitis within a month. No significant differences were found between the groups, except for higher bacterial eradication for the CS-3 10-day group (Table 3).

**Table 3:** Clinical course after completed PcV treatment for the 420 patients divided into groups based on Centor Score (3 or 4) and given treatment (Penicillin V 800 mg × 4 for 5 days or Penicillin V 1 g × 3 for 10 days)

	3-CS 1 g × 3, 10 days n = 104	3-CS 800 mg × 4, 5 days n = 104	p	4-CS 1 g × 3, 10 days n = 105	4-CS 800 mg × 4, 5 days n = 107	p
Patients with new sore throat related illness^a^ n (%)	3 (3)	8 (8)	0.19	3 (3)	7 (7)	0.17
Days to recurrence of sore throat-related illness^a^ (median)	1	1.5	0.82	1	2	0.12
Duration of the sore throat- related illness^a^ (median)	2	3	0.81	3	3	0.39
Bacterial eradication at follow-up visit n (%)	84 (81)	78 (75)	0.03	81 (77)	82 (77)	0.29
New acute pharyngotonsillitis within one month n (%)	5 (5)	10 (10)	0.20	6 (6)	13 (12)	0.10

^a^Based on patients’ diaries

Bacterial isolates from 40 patients without bacteriological eradication at the follow-up visit were *emm*-typed. 14 different *emm*-types were identified. The most common *emm*-types were 1, 4, 12, 89, and 28, constituting 71% of the tested isolates at inclusion. One isolate was not possible to classify. There were no differences in distribution of *emm*-types between the groups with CS-3 and CS-4 (p = 0.38). At follow-up, three isolates were not possible to classify. In 34/36 patients, we found the same *emm*-type at the follow-up visit as at the inclusion visit.

There was no significant difference in dropouts for side effect registration between the groups. Some patients reported side effects on day of inclusion (day 0): 20/420 diarrhoea; 56/420 nausea or vomiting; 11/420 rash; and 12/269 vaginal itching or discharge. Self-reported side effects were mainly diarrhoea, nausea, or vomiting and vaginal itching and discharge (Table 4). No significant differences between the groups were found.

**Table 4:** Self-reported adverse events according to the patients’ diaries divided into groups based on Centor Score (3 or 4) and given treatment (Penicillin V 800 mg × 4 for 5 days or Penicillin V 1 g × 3 for 10 days)

	Adverse events							
	CS-3 1 g × 3, 10 days n = 104		CS-3 800 mg × 4, 5 days n = 104		CS-4 1 g × 3, 10 days n = 105		CS-4 800 mg × 4, 5 days n = 107	
	No (%)	Duration (days)^a^	No (%)	Duration (days)^a^	No (%)	Duration (days)^a^	No (%)	Duration (days)^a^
Diarrhoea	29 (28)	2 (1–3)	20 (23)	1 (1–2.5)	25 (24)	2 (1–3)	22 (21)	2 (1–2.25)
Nausea or vomiting	21 (20)	2 (1–4.5)	11 (11)	1 (1–2)	11 (11)	1 (1–4)	12 (11)	2 (1.25–4.75)
Vaginal itching or discharge (women)	15 (21)	6 (4–8)	7 (10)	3 (3–5.75)	10 (16)	2 (3–10)	6 (9)	4 (1–5)
Rash	5 (5)	2(1–5)	5 (5)	3 (1–6)	6 (6)	3 (1–9)	1 (1)	3 (3–3)

^a^Median (interquartile range)

## Discussion

In this prospective clinical study of patients with pharyngotonsillitis with group A streptococci, patients with CS-3 as well as CS-4 who received PcV 800 mg × 4 for 5 days reported recovering earlier than those who received treatment with PcV 1 g × 3 for 10 days. In addition, throat pain as a single symptom was relieved earlier (1 day) and the period the patient used painkillers was shorter in patients with CS-4 who received PcV 800 mg × 4 for 5 days compared to those who received standard treatment with PcV 1 g × 3 for 10 days. For patients with CS-3, there were no differences in days with throat pain as a single symptom and in the use of pain killers between those who received PcV 800 mg × 4 for 5 days and those who received standard treatment 1 g × 3 for 10 days.

### Comparison with other studies

As far as we know, this is the first study comparing the clinical course of sore throat in patients with CS-3 and patients with CS-4 treated with PcV. Earlier studies have considered patients with CS-3 or CS-4 as one group when comparing different antibiotic regimes [10, 14].

Antibiotics reduce the duration of symptoms in sore throat [12, 15], but previous studies on PcV treatment for sore throat have come to different conclusions regarding long and short treatment with the same daily dosage [13, 14, 16–18]. The Descarte cohort study showed no major difference in duration of symptoms and number of re-consultations between patients treated with PcV for 5 days and 10 days. The Descarte study adjusted for severity of symptoms at inclusion, but patients with sore throat were included irrespective of CS and no etiologic testing for GAS was used [13]. In two randomized controlled trials where patients with GAS received PcV for either 5 or 10 days, more bacteriologic failures were found in the 5-day than in the 10-day treatment groups. However, in these studies, the CS was not used and the PcV daily dosages were lower than in our study [16, 17]. In an RCT by Zwart, where patients with CS-3 or -4 were considered one group, the regression of symptoms was faster in the 7-day than in the 3-day treatment group, but there were no differences in number of re-consultations [14].

A recently published review concluded that long-course PcV should remain the first line antibiotic for the management of patients with streptococcal pharyngitis [8]; however, most of the studies comparing the duration of treatment with PcV were based on a three dose per day regimen and compared long-term antibiotic therapy (10 days PcV) with short-term broad-spectrum antibiotics. In our previous study, we showed that PcV four times daily for 5 days was non-inferior in clinical cure to PcV three times daily for 10 days in patients with pharyngotonsillitis with CS-3 or -4 and diagnosed with GAS. The subgroup analyses indicated lower clinical cure with the 5-day regime in the CS-4 group. These results were based on data from the per-protocol population and the test of cure visit [10]. In the present study, data were based on the intention to treat population and self-reported data in patient diaries, so it measures a more patient-related outcome.

The present study shows that the shorter four dose treatment regime seems to be beneficial regardless of CS-3 or CS-4, and patients report a faster overall recovery from infection with the shorter (5 days) and more intense treatment. Also, the number of days with severe to moderate throat pain was reduced faster among those with CS-4 when taking antibiotics four times a day. This finding is also supported by the fact that duration of analgesic use was shorter in the CS-4 5-day group. One likely explanation for the efficacy of the 5-day treatment is the longer time above MIC due to more frequent dosage [19]. This result answers the research question raised in the Cochrane review [8]. Furthermore, we found no differences in re-consultations within a month between the four groups. The present study is not powered to identify differences in re-consultation rates, therefore it should be relevant to investigate in further studies. The different results from test of cure and diaries may also be due to new sore throat symptoms at the test of cure, symptoms that do not necessarily lead to a re-consultation.

In many studies investigating sore throat, symptom reduction is the main outcome, which from a clinical perspective seems relevant since GAS can be present in healthy persons [20, 21]. As far as we know, this is the first study that shows that patients with more symptoms (i.e., patients with CS-4) benefit the most from a shorter but more intensive treatment strategy. Although the benefits are not as great for the patients in the CS-3 group, they also experience a shorter disease period with the more intense 5-day treatment.

We do not know why the severity of the symptoms varies, but it has been debated if the *emm*-type of GAS is important [22]. The *emm*-types vary over time with age group, and some studies have found that specific *emm*-types are more common in pharyngitis [23, 24], but this could not be confirmed in others [25]. The same *emm*-type can be found in both invasive and non-invasive diseases [26]. In the present study, the *emm*-types are similar to the most frequently isolated *emm*-types from invasive cases in Sweden during the study period [27], a finding that indicates factors other than *emm*-types influence severity.

Our previous study found that a shorter but more intense treatment regimen of PcV led to fewer side effects and a shorter duration of side effects [10]. This finding could be explained by a shorter exposure of PcV. This pattern was the same when divided by CS. The finding that there were fewer side effects with shorter duration in the 5-day group further strengthens the 5-day treatment strategy. A longer treatment period also gives higher antibiotic selection pressure, which increases the risk of resistance in society [1].

Empirical evidence of antibiotic treatment for children with pharyngotonsillitis is scarce according to a Cochrane review [15]. Zwart et al., comparing the effect of penicillin for 3 days, 7 days, and a placebo in children with sore throat, found that penicillin treatment had no beneficial effect on the average duration of symptoms [18]. In our study, we found that there were no differences in self-reported recovery and severity of sore throat in children (6– ≤ 11 years) regardless of CS and treatment regimen. Although the study was not powered for subgroup analysis in children, it raises the question of how much children with sore throat benefit from antibiotic treatment.

Overall, the results indicate that there are several benefits with 4-dose regimen for 5 days compared to the Swedish standard treatment, 3-dose regimen for 10 days, both among patients with CS-3 and CS-4. The intense treatment reduces the amount of PcV for every treatment against pharyngotonsillitis, from 30 g PcV to 16 g. This means a yearly reduction of almost 50% in antibiotic pressure for this indication in Sweden.

### Strengths and limitations

This study examines everyday clinical practice as we used inclusion criteria in line with current treatment guidelines and dosing regimens according to modern knowledge of pharmacokinetics and pharmacodynamics. As the diaries had a high response rate, we gathered the patient’s own assessment of symptoms and not just the doctor’s assessment. Another strength is that children were included in the study because they are often treated with antibiotics for respiratory tract infections in primary healthcare [3, 28]. In addition we performed *emm*-typing on those not bacteriological eradicated at the follow-up visit. This enhances the generalizability of the study by showing that the GAS found in the patients in our study are similar to those circulating in the society.

A limitation is that not all GAS isolates were *emm*-typed, so we cannot say if the distribution is the same in the CS-3 and CS-4 groups. In addition, the patients were aware of which dose regimen they received and this could have affected the reporting. Another limitation is that some diaries were not complete, but there were no significant differences in missing data between the groups.

## Conclusion

Intense treatment with PcV 4 times per day for 5 days seems clinically beneficial compared to the Swedish standard treatment (PcV 3 times per day for 10 days) when treating CS-3 and CS-4 patients with GAS positive pharyngotonsillitis. Both groups experienced a faster overall self-reported recovery; for patients with CS-4, the intense treatment also shortened the period of throat pain. A reduction of 14 g PcV in every treatment, no difference in relapses, and side effects further strengthens the suggestion that the 4-dose regimen with 800 mg PcV for 5 days may be the future treatment strategy for GAS positive pharyngotonsillitis.

## Data Availability

The data sets generated and analysed during the current study are not publicly available due to Swedish legislation (the Personal Data Act) but are available from the corresponding author on reasonable request.

## Abbreviations

GAS: Group A streptococci
CS: Centor Score
PcV: Phenoxymethylpenicillin
RADT: Rapid antigen detection test
ESCMID: European Society of Clinical Microbiology and Infectious Diseases
